# Shock and Awe: The Tactical Trade-Offs of Impella^®^ Versus Intra-Aortic Balloon Pump in Takotsubo Cardiomyopathy

**DOI:** 10.3390/reports8020043

**Published:** 2025-04-02

**Authors:** Ajay Saraf, Amit Goyal

**Affiliations:** 1 Internal Medicine Residency, Boonshoft School of Medicine, Wright State University, Dayton, OH 45435, USA; 2 Cardiovascular Medicine, Premier Heart Institute, Boonshoft School of Medicine, Wright State University, Dayton, OH 45435, USA

**Keywords:** takotsubo cardiomyopathy, cardiogenic shock, mechanical circulatory support, intra-aortic balloon pump, Impella, stress-induced cardiomyopathy

## Abstract

**Background and Clinical Significance:** Takotsubo cardiomyopathy (TCM), an acute stress-induced left ventricular dysfunction, stems from catecholaminergic surges leading to transient myocyte stunning, calcium overload, and microvascular dysregulation. Although most cases resolve spontaneously, roughly 10% deteriorate into fulminant cardiogenic shock, warranting mechanical circulatory support (MCS). Impella^®^ provides direct transvalvular LV unloading but carries elevated risks of hemolysis, vascular compromise, and thrombogenicity. Conversely, the intra-aortic balloon pump (IABP) enhances diastolic coronary perfusion and marginally reduces afterload via counterpulsation, albeit with less potent LV decompression. Optimal MCS selection in TCM-associated shock therefore hinges on balancing hemodynamic benefits against procedural morbidity. **Case Presentation:** A 72-year-old female with coronary artery disease, paroxysmal atrial fibrillation (status post–left atrial appendage occlusion), and stage 3 chronic kidney disease presented with anterior ST-segment elevations (V2–V4) and troponin I >1000 ng/L, progressing rapidly to cardiogenic shock and respiratory failure. Coronary angiography revealed mild luminal irregularities, while echocardiography demonstrated severely reduced ejection fraction (5–10%) with characteristic apical ballooning. Refractory elevations in pulmonary capillary wedge pressure, despite escalating inotropes and vasopressors, prompted IABP insertion for partial LV offloading. Over one week, her ejection fraction improved to 35%, facilitating weaning from pressor support, extubation, and discharge on guideline-directed medical therapy. **Conclusions:** In TCM complicated by shock, meticulous MCS selection is paramount. Although Impella confers more robust unloading, heightened device-related complications may be unjustified in a largely reversible disease. IABP can sufficiently stabilize hemodynamics, enable myocardial recovery, and mitigate morbidity, underscoring the importance of individualized decision-making in TCM-related shock. Importantly, no trial has shown that MCS confers a proven long-term mortality benefit beyond initial hemodynamic rescue.

## 1. Introduction and Clinical Significance

Takotsubo cardiomyopathy (TCM), often termed stress-induced cardiomyopathy or apical ballooning syndrome, is an acute, transient left ventricular systolic dysfunction precipitated by neurohormonal surges that exert deleterious effects on the myocardium in the absence of significant epicardial coronary obstruction [1]. Although the pathophysiology is not fully understood, exaggerated catecholaminergic activity can induce microvascular ischemia, metabolic derangements, and regional contractile dyskinesis [2]. Approximately 6–10% of TCM patients progress to fulminant cardiogenic shock, requiring emergent hemodynamic support [3,4].

In TCM complicated by shock, mechanical circulatory support (MCS) can be lifesaving in the acute setting. Impella^®^ delivers robust transvalvular LV unloading but is associated with increased hemolysis, vascular complications, and bleeding [5,6]. In contrast, the IABP primarily reduces afterload and augments coronary perfusion, yet entails fewer device-related adverse events [5,7]. Notably, while MCS may be critical for reducing acute mortality, it has not been shown to improve chronic outcomes in TCM; long-term morbidity and mortality are most strongly influenced by guideline-directed medical therapy [4,8]. Here, we present the case of a 72-year-old female with severe TCM-related cardiogenic shock successfully stabilized via IABP support, highlighting the device’s capacity to restore hemodynamic equilibrium in a condition that can recover once the acute insult abates and guideline directed medical therapy is initiated.

## 2. Case Presentation

### 2.1. Patient Profile and Medical History

A 72-year-old female with a long-standing history of coronary artery disease, paroxysmal atrial fibrillation (status post left atrial appendage occlusion via Watchman^®^), and chronic kidney disease stage 3 presented to the Emergency Department with severe substernal chest pain radiating to the left arm, accompanied by progressive dyspnea. Her outpatient medical regimen included beta-blockade and high-intensity statin. She reported no recent hospital admissions prior to the current admission.

### 2.2. Initial Presentation

On arrival, the patient demonstrated hypotension (systolic blood pressure < 90 mmHg), tachycardia (~120 bpm), and marked respiratory distress. A 12-lead electrocardiogram displayed ST-segment elevations in leads V2–V4 and reciprocal depressions in the inferior leads, raising concern for an acute anterior ST-elevation myocardial infarction. Laboratory data revealed a significantly elevated troponin I level (>1000 ng/L), suggesting substantial myocardial injury.

### 2.3. Diagnostic Workup

1. Coronary Angiography: Emergent cardiac catheterization identified only mild non-obstructive coronary lesions (<30% stenoses), effectively excluding a culprit epicardial lesion. Left ventriculography demonstrated systolic ballooning of the apical segments with basal hyperkinesis.

2. Transthoracic Echocardiogram: Echocardiography confirmed a severely depressed ejection fraction of approximately 5–10%, pronounced apical hypokinesis, and the absence of significant valvular disease, supporting a diagnosis of Takotsubo cardiomyopathy.

3. Hemodynamic Monitoring: Placement of a pulmonary artery catheter revealed markedly elevated pulmonary capillary wedge pressures (~28 mmHg) and a reduced cardiac index (<1.8 L/min/m^2^), confirming cardiogenic shock physiology.

### 2.4. Management and Clinical Course

Despite the initiation of intravenous vasopressors and inotropes, the patient’s hemodynamic status remained precarious, with persistently low mean arterial pressures and rising lactate levels. In light of TCM’s propensity for spontaneous myocardial recovery and the desire to limit complications such as hemolysis and vascular injury, the clinical team opted for intra-aortic balloon pump support rather than more aggressive mechanical circulatory support.

Following IABP insertion, the patient demonstrated steady improvements in mean arterial pressure, urine output, and mixed venous oxygen saturation, allowing cautious weaning of vasopressor infusions over 72 h. Repeat echocardiograms documented progressive recovery of left ventricular contractility, with ejection fraction increasing from 5–10% on admission to approximately 20% by Day 3. Diuretic therapy was intensified during this period to manage ongoing volume overload.

By Day 7, the patient’s ejection fraction approached 35%, enabling discontinuation of IABP assistance and de-escalation of all inotropic and vasoactive agents. Her renal function returned to baseline, and she was extubated shortly prior to discharge from the ICU.

### 2.5. Outcome and Discharge

Ultimately, the patient was transferred out of the intensive care unit after demonstrating stable hemodynamics without mechanical support. She was discharged on an optimized heart failure regimen, including beta-blockers and an angiotensin-converting enzyme inhibitor, along with high-intensity statin therapy.

This case illustrates that IABP can provide sufficient hemodynamic stabilization in severe TCM-related shock without incurring the elevated complication profile often associated with more aggressive device-based interventions.

## 3. Discussion

### 3.1. Pathophysiology of TCM and Progression to Shock

Takotsubo cardiomyopathy is frequently referred to as stress-induced cardiomyopathy or apical ballooning syndrome and is characterized by regional left ventricular (LV) systolic dysfunction in the absence of obstructive coronary artery disease [1]. It typically arises from a transient surge in catecholamines that induces microvascular dysregulation, hyperdynamic basal contraction, and akinesis of the mid-to-apical segments [2]. Although most TCM cases follow a relatively benign clinical course with recovery aided by standard heart failure therapy, approximately 6–10% deteriorate into cardiogenic shock [2]. In these fulminant presentations, the overwhelming catecholaminergic state leads to severely depressed cardiac output, elevated filling pressures, and systemic hypoperfusion, usually requiring emergent hemodynamic stabilization.

### 3.2. Hemodynamic Considerations in TCM Shock

Unlike ischemic cardiogenic shock, TCM shock often exhibits a discordant contraction pattern, with profoundly akinetic apical segments and compensatory basal hyperkinesis [1]. This regional dysfunction can exacerbate intraventricular pressure differentials, occasionally precipitating left ventricular outflow tract obstruction. Moreover, the absence of culprit epicardial lesions means that inotropic agents aimed at boosting contractility may paradoxically worsen myocardial insult by further increasing catecholaminergic drive [2]. Instead, physiologic optimization typically centers on reducing afterload to aid forward flow, diuresis to alleviate pulmonary congestion, and, in severe refractory cases, mechanical circulatory support to bridge the stunning period [3].

### 3.3. MCS Selection in TCM Shock: Intra-Aortic Balloon Pump vs. Impella^®^

Two principal percutaneous strategies for mechanical circulatory support have been explored in TCM: the intra-aortic balloon pump, IABP, and the Impella^®^ device. The intra-aortic balloon pump employs diastolic counterpulsation, inflating in diastole to augment coronary perfusion and deflating just before systole to reduce afterload [4]. Although its unloading capacity is more modest than that of Impella^®^, the intra-aortic balloon pump carries fewer device-related complications, including significantly lower rates of hemolysis, major bleeding, and vascular compromise [5]. This safety profile may be especially advantageous in TCM, where recovery can be achieved with combined mechanical support (for acute stabilization) and guideline-directed medical therapy (for long-term benefit).

Impella^®^ provides direct left ventricular decompression via a microaxial flow pump that traverses the aortic valve, offering robust augmentation of cardiac output [5]. In principle, this degree of offloading can accelerate recovery by reducing wall stress; however, clinical data in cardiogenic shock, encompassing both ischemic and non-ischemic etiologies, often demonstrate elevated complication rates—most notably hemolysis, limb ischemia, and bleeding—without a definitive mortality benefit over the intra-aortic balloon pump [5]. Hence, while Impella^®^ may be necessary in the most severe TCM presentations, such as profound hypotension, severe left ventricular outflow tract obstruction, or biventricular compromise, the intra-aortic balloon pump remains a prudent first-line device in many instances of TCM shock.

### 3.4. Clinical Outcomes and Lessons from Registries

Evidence from registries and observational cohorts has reshaped the perception that TCM is always benign. Approximately 10% of patients develop shock, often mirroring the morbidity of acute coronary syndromes [1]. In the German–Italian–Spanish, registry, intra-aortic balloon pump use in TCM shock did not confer a statistically significant mortality reduction compared to no mechanical support, reflecting both the severity of illness in those requiring support and potential selection bias [4]. Nonetheless, case reports and smaller series consistently demonstrate that the intra-aortic balloon pump can stabilize hemodynamics sufficiently to enable myocardial recovery, with ejection fractions frequently improving from <20% to near normal over days to weeks [3].

Impella^®^ has also been applied in TCM shock, with small multicenter analyses describing rapid unloading and early extubation in certain subsets of patients [8]. However, no prospective, randomized trial has demonstrated the superiority of MCS over medical therapy in terms of long-term mortality or morbidity in TCM, and it remains unclear whether the more robust unloading of Impella^®^ translates into superior survival or faster functional restoration specifically in TCM [5]. While MCS can be pivotal for reducing acute mortality, long-term outcomes remain contingent on sustained heart failure therapy and optimization of comorbid conditions. Given that TCM’s hallmark is reversibility, the elevated risk profile of more aggressive mechanical circulatory support must be carefully weighed against the possibility of partial myocardial recovery within a short interval.

### 3.5. Comparison with Other Cardiogenic Shock Etiologies

Whereas TCM shock arises from transient myocardial stunning without an occlusive lesion, ischemic cardiogenic shock typically reflects irreversible necrosis and requires urgent revascularization [6]. The reversibility of TCM explains why conservative support approaches, including the intra-aortic balloon pump, may suffice, whereas in severe ischemic shock, more robust support such as Impella^®^ or venoarterial extracorporeal membrane oxygenation may be necessary to sustain failing myocardium while pursuing interventions such as percutaneous coronary intervention or bypass surgery [6]. Moreover, TCM typically resolves once the inciting neurohormonal storm abates—provided medical therapy is thereafter optimized—distinguishing it from myocarditis or dilated cardiomyopathy, where persistent inflammation or structural remodeling can preclude complete recovery.

### 3.6. Future Directions

Although multiple registries have improved our understanding of TCM shock, key questions remain. Prospective, controlled trials comparing the intra-aortic balloon pump and Impella^®^ in TCM shock are needed to clarify best practices, particularly for patients with extremely low ejection fraction or coexisting left ventricular outflow tract obstruction [5]. Novel support strategies, including hybrid devices with lower profiles or more biocompatible materials, may further minimize procedural hazards. Enhanced hemodynamic monitoring—such as real-time measurement of left ventricular pressure–volume loops—could guide more nuanced device deployment and weaning. Additionally, closer examination of TCM subtypes, for example, apical vs. midventricular ballooning, might clarify which patients derive maximal benefit from advanced mechanical circulatory support.

Better delineation of TCM’s neuropsychiatric triggers, the contribution of microvascular ischemia, and the effects of proinflammatory mediators could ultimately enable targeted therapies that mitigate myocardial stunning more directly. Large-scale, longitudinal studies remain necessary for refining risk stratification tools to identify patients at imminent risk of shock who may benefit from earlier support. As this field evolves, integrating pharmacologic, psychologic, and device-based strategies holds promise for enhancing survival and long-term quality of life in patients with TCM-related cardiogenic shock.

## 4. Conclusions

Takotsubo cardiomyopathy, though often transient, can precipitate severe cardiogenic shock in a minority of patients, underscoring the importance of early recognition and prompt intervention. Mechanical circulatory support can stabilize hemodynamics while the stunned myocardium recovers, and in many cases, the intra-aortic balloon pump provides a prudent first-line option given its relatively low complication profile and sufficient unloading capacity for a self-limiting disease. More aggressive devices, such as Impella^®^, may prove necessary for selecting patients with profound hemodynamic compromise, but the potential risks, including hemolysis and vascular injury, must be weighed against TCM’s intrinsic capacity for improvement. Crucially, while MCS has not been shown to enhance chronic survival or reduce long-term morbidity in TCM, it remains critical for short-term rescue. Overall, these observations highlight the need for individualized MCS selection, rigorous hemodynamic monitoring, and attention to any complicating factors such as left ventricular outflow tract obstruction. Continued research, ideally through prospective trials comparing different support modalities, may ultimately refine best practices and optimize outcomes in TCM-related shock.

## Data Availability

Data supporting the findings of this study are not publicly available due to privacy and ethical restrictions. These restrictions are in place to protect the confidentiality of the study participants and adhere to applicable data protection regulations.

## Abbreviations

The following abbreviations are used in this manuscript:

IABP	Intra-aortic balloon pump
LV	Left ventricle
LVOT	Left ventricular outflow tract
MCS	Mechanical circulatory support
TCM	Takotsubo cardiomyopathy
